# Contribution of astrocytes to neurovascular coupling in the spinal cord of the rat

**DOI:** 10.1186/s12576-021-00800-6

**Published:** 2021-05-28

**Authors:** Thierry Paquette, Mathieu Piché, Hugues Leblond

**Affiliations:** 1 Department of Anatomy, Université du Québec À Trois-Rivières, 3351 Boulevard des Forges, C.P. 500, Trois-Rivières, QC G9A 5H7 Canada; 2 CogNAC Research Group, Université du Québec À Trois-Rivières, Trois-Rivières, QC G9A 5H7 Canada

**Keywords:** Astrocyte, Fluorocitrate, Spinal cord, Neurovascular coupling, Blood flow, Local field potential

## Abstract

Functional magnetic resonance imaging (fMRI) of the spinal cord relies on the integrity of neurovascular coupling (NVC) to infer neuronal activity from hemodynamic changes. Astrocytes are a key component of cerebral NVC, but their role in spinal NVC is unclear. The objective of this study was to examine whether inhibition of astrocyte metabolism by fluorocitrate alters spinal NVC. In 14 rats, local field potential (LFP) and spinal cord blood flow (SCBF) were recorded simultaneously in the lumbosacral enlargement during noxious stimulation of the sciatic nerve before and after a local administration of fluorocitrate (*N * = 7) or saline (*N*  = 7). Fluorocitrate significantly reduced SCBF responses (*p * < 0.001) but not LFP amplitude (*p * = 0.22) compared with saline. Accordingly, NVC was altered by fluorocitrate compared with saline (*p * < 0.01). These results support the role of astrocytes in spinal NVC and have implications for spinal cord imaging with fMRI for conditions in which astrocyte metabolism may be altered.

## Introduction

The non-invasive assessment of spinal cord functions by neuroimaging methods has contributed to the advancement of basic and clinical neuroscience [34, 48]. Recent studies have shown that spinal cord activity induced by painful stimuli can be assessed by functional magnetic resonance imaging (fMRI) [38, 43]. However, these fMRI methods rely on the blood oxygen level-dependent signal (BOLD), a hemodynamic response used to infer the underlying neuronal activity [19], a link referred to as the neurovascular coupling (NVC). Thus, it is essential to understand NVC properties and mechanisms to improve spinal cord fMRI data interpretation and validity. Although, previous studies have examined spinal NVC [27, 28, 33], its mechanisms remain unclear.

In the brain, the NVC is regulated by multiple mechanisms and cell types that are described as the neurovascular unit [8, 17, 25]. In addition to neurons and pericytes, astrocytes contribute to blood flow regulation, acting as a mediator between neuronal populations and their surrounding blood vessels [4, 13, 23, 36, 41, 51]. These findings are corroborated with supraspinal BOLD fMRI experiences where they manipulate astrocyte activity with optogenetic [42], pharmacological [15] and stimulation paradigm [7]. In response to the release of glutamate by neurons, intracellular Ca^2+^ increases in astrocytes, which in turn causes the release of vasodilator agents such as epoxyeicosatrienoic acids and prostaglandin E2 [1, 13, 23, 51]. When the locus coeruleus (LC) is activated, the release of noradrenaline also increases intracellular Ca^2+^ in astrocytes [3], which implies that the NVC unit may produce different responses depending on the origin of neuronal activity, stimulation characteristics, or brain-state [47].

In spite of the critical role of the neurovascular unit in cerebral blood flow regulation, the components of the spinal cord neurovascular unit have never been examined. This seems essential, especially for the validation of spinal fMRI, because some discrepancies have been reported between spinal and cerebral NVC [11, 21, 28, 33, 45]. In contrast to cortical NVC, spinal NVC is not affected by isoflurane anesthesia and by large fluctuations in mean arterial pressure during nociceptive stimulation [28, 33]. In addition, astrocytes contribute to autoregulation during arterial pressure changes [12] and they are sensitive to isoflurane [35] in the brain. Thus, their contribution to NVC regulation in the spinal cord may differ, but this remains unclear.

In previous studies, fluorocitrate has been used to determine the roles of astrocytes in the central nervous system and to test neuronal function in the absence astrocyte activity [24, 50]. Astrocytes selectively uptake fluorocitrate, which inhibits aconitase, an enzyme in the Krebs cycle, which in turn inhibits astrocyte metabolic activity [6, 9, 29]. The inhibition is observable 30 min after the application of fluorocitrate [16] with a maximum effect after 4 h [9, 29]. Depending on fluorocitrate concentration, astrocyte inhibition may be reversible [29]. At high doses, however, (over 2 nmol), the effects are irreversible and even lead to degeneration of neurons [29].

The objective of the present study was to examine the contribution of astrocytes to the spinal cord NVC during noxious stimulation. Based on the critical role of astrocytes in the neurovascular unit of the brain, we hypothesized that astrocyte inhibition by fluorocitrate would reduce SCBF responses without affecting neuronal activity, therefore altering the NVC.

## Methods

### Animals and surgical procedures

Experiments were performed on 14 male Wistar rats (body weight: 320–450 g; age: 18–22 weeks; Charles River Laboratories, Saint-Constant, Québec, Canada). Animals were housed in the animal facilities of Université du Québec à Trois-Rivières with access to food and water ad libitum. A light–dark cycle of 14–10 h was maintained. All animals were in good health on the day of the experiment. The 14 rats were assigned to the fluorocitrate group (*N * = 7) or the control group (*N*  = 7). Surgical procedures were conducted under isoflurane anesthesia (2.5%) as described previously [28, 33]. The depth of anesthesia was confirmed by a stable mean arterial pressure (MAP) and was assessed routinely by paw pinching (withdrawal reflex). Rectal temperature was monitored and maintained at 37.5 ± 0.5 °C with a custom-made temperature control system. The animal was ventilated mechanically (SAR-830/P 123 Ventilator, CWE Inc., Ardmore, PA, USA) using a tracheal cannula. The ventilation parameters were adjusted to maintain an end-tidal CO_2_ around 3.0% (Covidien Capnostream 35 Monitor, Covidien, Dublin, Ireland). The MAP was recorded continuously using a cannula inserted into the right carotid artery and connected to a pressure transducer (Harvard Apparatus, Holliston, MA, USA). For all animals, MAP remained above 70 mmHg during the entire experiment. Intravenous injections were administered in the catheterized right jugular vein. The left sciatic nerve was dissected for electrical stimulation and a laminectomy was performed between T12 and L1 to access the lumbar enlargement for recordings.

### Experimental protocol

After surgical procedures were completed, the multi-electrode was inserted in the spinal cord, the laser-Doppler probe was placed on the surface of the spinal cord and isoflurane anesthesia was lowered to 1.5%. After 45 min of rest, continuous recording of LFP and SCBF began. The stimulation protocol consisted of a series of electrical stimuli at graded intensity, ranging between 0.1 and 9.6 mA. The series of stimuli was applied 5 times, once before fluorocitrate or saline administration, and 1 h, 2 h, 3 h and 4 h after their administration. The order of the 8 stimulus intensities was randomized between animals to avoid sequence order effects, but the 5 series were delivered using the same sequence.

### Drug preparation and administration

The fluorocitrate solution was prepared as described by Paulsen et al. [29]. Eight mg of d,l-fluorocitric acid, Ba^3^ salt (Sigma-Aldrich, Oakville, Canada) was dissolved in 1 ml of 0.1 M HCl. Three drops of 0.1 M Na_2_S0_4_ were added to precipitate Ba^2 +^ . Na_2_HPO_4_ was then added (2 ml of 0.1 M) and the suspension was centrifuged at 1000*g* for 5 min. The supernatant was diluted with 0.9% NaCl to the final concentration and the pH was adjusted to 7.4. The solution (0.1 ml at 0.01 nmol/µl) was applied directly on the surface of the spinal cord between T12 and L1 within a pool made of petroleum jelly. This fluorocitrate concentration was selected based on a previous study showing that a concentration of 1 nmol progressively inhibits astrocytes without affecting neurons [29]. After confirming proper anesthesia, gallamine triethiodide (20 mg/kg) was injected through the jugular cannula 20 min before the stimulation protocol began to immobilize the animal and ensure stable recordings.

### Local field potentials

A 16-channel microelectrode (Model A1 × 16–10 mm-200-100-177, Neuronexus Technologies Inc., Ann Arbour, Michigan, USA) was inserted into the left lumbar enlargement using a micromanipulator (Model 960, David Kopf Instruments, Tujunga, CA, USA). The microelectrode position was set where electrical stimulation of the sciatic nerve produced multiunit responses in most of the superficial channels (Smartbox, Neuronexus Technologies Inc., Ann Arbour, Michigan, USA; band pass: 300–3000 Hz, gain: 192 V/V). Signal was sampled at 20 kHz with a broad band (1–10,000 Hz, gain: 192 V/V) (Smartbox, Neuronexus Technologies Inc., Ann Arbour, Michigan, USA) and recorded on a personal computer for offline analyses. Offline signal processing included resampling at 5 kHz and band pass filtering (1–300 Hz) to obtain LFPs. For each rat, the channel that showed LFP of the greatest amplitude in the superficial layers of the spinal cord was selected for further analyses (see “Data analyses”).

### Spinal cord blood flow

Spinal cord blood flow (SCBF) was recorded with a laser-Doppler probe (Micro-needle probe TSD145, Biopac systems, Goleta, CA, USA). The probe was placed on the spinal cord surface, as close as possible from the microelectrode, carefully avoiding large blood vessels. SCBF was sampled at 100 Hz with a time constant of 3 s and was recorded on a computer for offline analyses (Power 1401 acquisition system, Cambridge Electronic Design, Cambridge, UK).

### Fluorocitrate effect on vasculature

To test the effect of fluorocitrate on smooth muscle cells of the vasculature, we applied 1 nmol of fluorocitrate on arterioles located in the neck muscles where there is no astrocyte. The vessel diameter was recorded with a HD camera connected to a binocular, with an objective of 40 ×. The diameter of the arterioles (*N*  = 9 in two rats) was analyzed offline with 14 time points (every 20 min) over a period of 4 h (ImageJ software). On three of these arterioles, we measured blood flow fluctuations using the same laser-Doppler probe over 4 h.

### Electrical stimulation of the sciatic nerve

Electrical stimulation was applied to the sciatic nerve using a custom-made bipolar hook electrode and a constant-current stimulator (Model DS7A, Digitimer Ltd, Welwyn Garden city, UK). Stimulation consisted of 10-s trains of 1-ms pulses delivered at 5 Hz. The inter-train interval of 55 s allowed physiological parameters to return to baseline values. Intensity was set for each train at 0.1, 0.15, 0.3, 0.6, 1.2, 2.4, 4.8, or 9.6 mA.

### Data analyses

LFP, SCBF and MAP data were analyzed using Spike2 software (Cambridge Electronic Design, Cambridge, UK, version 6.15). For the quantification of LFP amplitude, the peak-to-peak value of averaged potentials (50 responses induced by the stimulus train of 10 s at 5 Hz) was extracted with a custom-made Spike2 script within a 50-ms window following stimulus onset. For SCBF and MAP changes, the onset-to-peak value was extracted for each stimulus intensity within a 30-s window and response amplitude was calculated as the change relative to the mean signal value for the 30 s artifact-free baseline preceding stimulus onset. MAP responses were calculated as the raw change (mm Hg) while SCBF responses were calculated as percent change. The SCBF and LFP values were transformed into *T* scores for standardization to calculate the neurovascular coupling, indexed by the SCBF/LFP ratio.

### Statistical analyses

All results are expressed as means ± SEM. Statistical analyses were performed with Statistica (TIBCO software Inc. 2017. Statistica version 13) with a significance threshold of *p * ≤ 0.05. The effect size is reported as partial eta-squared (*η*^*2*^_*p*_). The sample size was estimated with a power calculation using G*Power v3.0.10 (G*Power, Kiel, Germany). Based on a conservative effect size ranging between *η*^*2*^_*p*_ = 0.1–0.2, an α of 0.05, and a power of 0.8, we obtained a required sample size ranging between 4 and 7 animals per group. To obtain the most reliable and reproducible results, we used 7 animals per group.

MAP, SCBF, LFP amplitude as well as neurovascular coupling ratios were compared between groups (fluorocitrate vs saline) using Greenhouse–Geisser corrected mixed ANOVAs, with intensity (0.1, 0.15, 0.3, 0.6, 1.2, 2.4, 4.8, 9.6 mA) and time (baseline, 1 h, 2 h, 3 h, 4 h) as repeated factors. Significant effects were then decomposed with planned contrasts to test a priori hypotheses.

## Results

### Mean arterial pressure

**Fig. 1:**
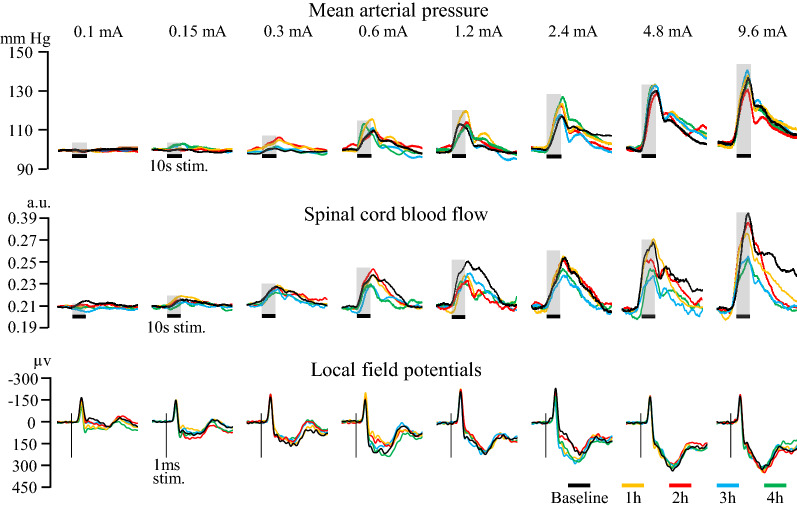
Individual example of physiological recordings. Representative recordings of MAP, SCBF and LFP responses for the eight stimulus intensities every hour, in one rat that received fluorocitrate. MAP and SCBF responses are a response to a single stimulus. LFP responses represent a mean of 50 responses. The stimulation produced intensity-dependent increases in MAP that remained stable over time and unaffected by fluorocitrate. The stimulation also produced intensity-dependent increases in SCBF. However, response amplitude progressively decreased over time due to the exposure to fluorocitrate. As for LFP, the expected negative and positive deflections were observed with an intensity-dependent amplitude. The peak-to peak amplitude of these deflections was stable over time and unaffected by fluorocitrate. The shaded areas with a horizontal black line (MAP and SCBF) indicate stimulus duration. The vertical grey bars (LFP) indicate the stimulus delivery (one of the 1 ms pulses from the train of 50 pulses)

**Fig. 2:**
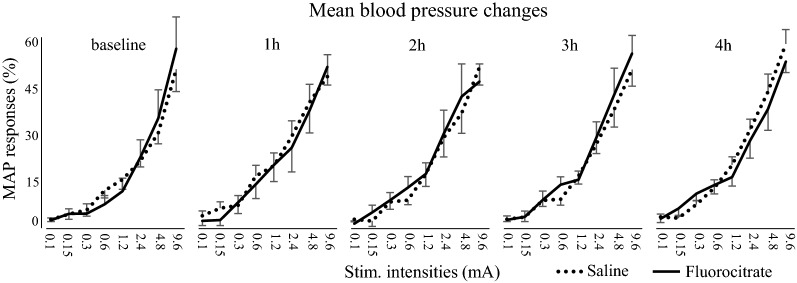
Sciatic nerve stimulation produced intensity-dependent increases in mean arterial pressure (*p*  <  0.001). Both groups showed similar blood pressure responses between intensities and over time (*p * <  0.8). *N * =  7 for each group. Error bars indicate SEM

An individual example of MAP responses is presented in Fig. 1 and the average MAP changes are presented in Fig. 2. Sciatic nerve stimulation produced intensity-dependent MAP increases (*F*_7,84_ = 125.0, *p*  < 0.001; *η*^*2*^_*p*_ = 0.91). These changes were stable and comparable between baseline and other time points (*F*_4,48_ = 1.27, *p*  = 0.29; *η*^*2*^_*p*_ = 0.09). Moreover, MAP changes were not significantly different between groups over time (*F*_4,48_ = 0.27, *p*  = 0.89; *η*^*2*^_*p*_ = 0.02) or across stimulation intensities (*F*_7,84_ = 0.08, *p*  = 1; *η*^*2*^_*p*_ = 0.01). This indicates that fluorocitrate did not alter MAP changes. The stability of MAP changes over 4 h also confirms that physiological conditions remained stable during the entire experiment.

### Spinal cord blood flow

**Fig. 3:**
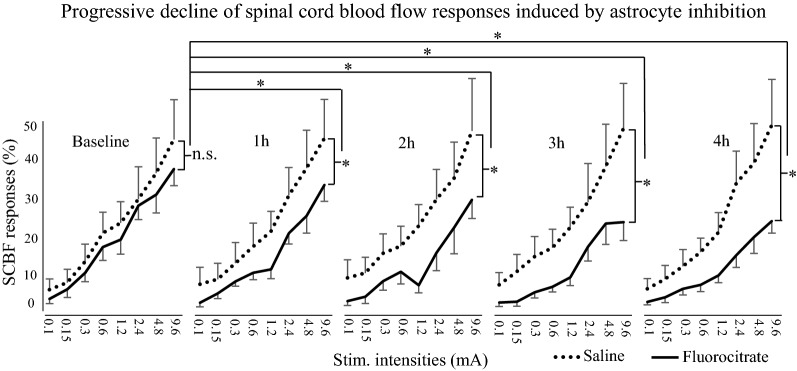
Electrical stimulation of the left sciatic nerve evoked intensity-dependent SCBF responses (*p* <  0.001). These increases were significantly different between the fluorocitrate (solid line) and control (dotted line) group over time (*p * <  0.001). *N*  =  7 for each group. Error bars indicate SEM. *p  <  0.05

SCBF responses are presented in Fig. 3 and an individual example is also presented in Fig. 1. Electrical stimulation produced intensity-dependent SCBF increases (*F*_7,84_ = 46.5, *p*  < 0.001; *η*^*2*^_*p*_ = 0.79). These increases were significantly different between groups over time (*F*_4,48_ = 6.6, *p * < 0.001; *η*^*2*^_*p*_ = 0.36). Planned contrasts revealed that the effects of fluorocitrate on the SCBF changes were significantly different compared with saline at 1 h, 2 h, 3 h and 4 h compared with the baseline (all *p*  < 0.05). As expected, the effect of fluorocitrate progressively increased, as shown by greater reduction of SCBF changes with time (linear trend: *p * < 0.001), while no such trend was observed with saline (*p * = 0.6). On average, for all intensities combined, fluorocitrate reduced SCBF changes by 23.8 ± 9.4% at 1 h, by 31.2 ± 14.1% at 2 h, by 40.2 ± 11.9% at 3 h and by 38.3 ± 10.5% at 4 h compared with the baseline. In contrast, SCBF changes in the saline group were stable over time with limited fluctuations (1.2 ± 11.4% at 1 h, 9.7 ± 20.0% at 2 h, 5.8 ± 15.4% at 3 h and 1.0 ± 13.5% at 4 h).

Baseline SCBF recorded over 30 s before every stimulation was comparable between groups over time (*F*_4,48_ = 0.99, *p*  = 0.4; *η*^*2*^_*p*_ = 0.07). On average, in comparison to baseline values recorded before fluorocitrate administration, baseline SCBF varied by 3.2 ± 8.0% at 1 h, by 1.9 ± 9.9% at 2 h, by 3.5 ± 11.1% at 3 h and by − 1.5 ± 11.0% at 4 h. Comparable changes were observed in the saline group: 0.9 ± 6.8% at 1 h, by 4.8 ± 5.9% at 2 h, by 1.6 ± 7.4% at 3 h and by 3.8 ± 5.2% at 4 h.

### Spinal cord local field potentials

**Fig. 4:**
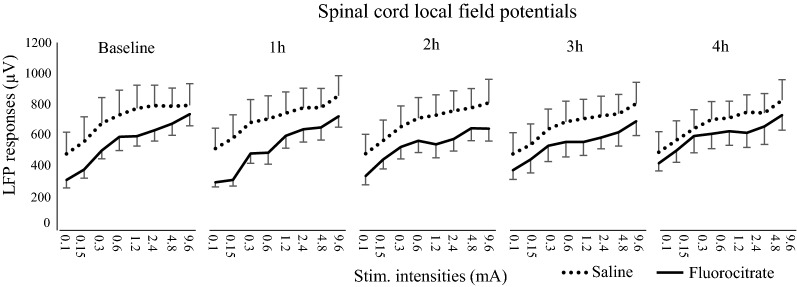
Electrical stimulation evoked intensity-dependent LFP (*p * <  0.001). LFP amplitude of fluorocitrate (solid line) and saline (dotted line) groups were similar over time (*p*  =  0.22). *N*  =  7 for each group. Error bars indicate SEM

Results for LFP amplitude are presented in Fig. 4 an individual example is also presented in Fig. 1. LFP amplitude increased with stimulus intensity (*F*_7,84_ = 20.5, *p*  < 0.001; *η*^*2*^_*p*_ = 0.02). However, LFP amplitude was not significantly different between groups over time (*F*_4,48_ = 1.5, *p*  = 0.22; *η*^*2*^_*p*_ = 0.1), indicating that astrocyte inhibition by fluorocitrate did not modulate neuronal activity.

### Spinal cord neurovascular coupling

**Fig. 5:**
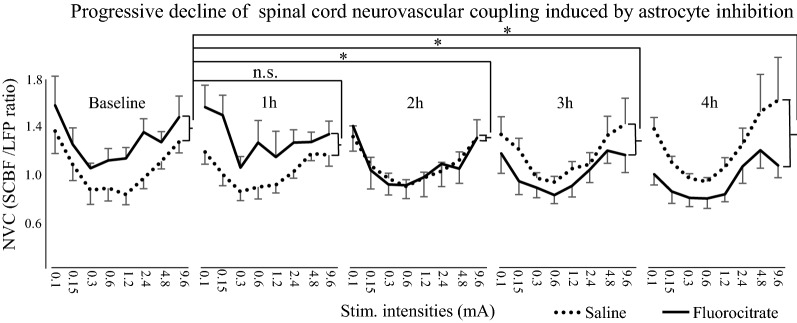
The neurovascular coupling was calculated as the SCBF/LFP ratio. The coupling was significantly different between the fluorocitrate (solid line) and control (dotted line) group over time (*p * <  0.01). The progressive decrease of the neurovascular coupling in the fluorocitrate group was significant from 2 h and afterward following fluorocitrate administration (*p * <  0.04). Error bars = SEM, *p  <  0.05

Results for NVC are presented in Fig. 5. NVC was significantly different between groups over time (*F*_4,48_ = 4.8, *p * < 0.01; *η*^*2*^_*p*_ = 0.28). Planned contrasts revealed that fluorocitrate significantly reduced NVC at 2 h, 3 h and 4 h compared with the baseline (all *p*  ≤ 0.04) consistent with the alteration of SCBF and the lack of effect on LFP amplitude. Like SCBF, NVC progressively decreased with time (linear trend: *p * = 0.04), while no such trend was observed with saline (*p*  = 0.17).

### Fluorocitrate effects on vasculature

To confirm the lack of effects of fluorocitrate on vascular smooth muscles, fluorocitrate was applied on arterioles located in the neck muscles which are devoid of surrounding astrocytes. Arterioles diameter did not change significantly over 4 h for the 14 time points (*F*_13,104_ = 1.2, *p * = 0.3; *η*^*2*^_*p*_ = 0.12). On average, in comparison to the baseline diameter measured before fluorocitrate administration, the diameter varied by 1.6 ± 1.0% at 1 h, by 1.2 ± 0.9% at 2 h, by 0.3 ± 1.6% at 3 h and by 1.0 ± 0.6% at 4 h. Arterial blood flow of three of these arterioles also remained stable over time after fluorocitrate administration: Friedman ANOVA (*χ*^2^(4) = 2.7, *p*  = 0.6). On average, in comparison to the baseline arterial blood flow measured before fluorocitrate administration, the blood flow varied by − 0.1 ± 3.9% at 1 h, by 2.1 ± 2.2% at 2 h, by 2.9 ± 3.0% at 3 h and by − 0.8 ± 4.9% at 4 h. These results demonstrate that fluorocitrate does not act on the vascular tissue.

## Discussion

The present results confirm our hypothesis and show that fluorocitrate administration decreases SCBF responses without affecting neuronal activity. Indeed, astrocyte inhibition by fluorocitrate reduced SCBF responses by up to 40% while neuronal activity was unaffected. This indicates that astrocytes contribute to the regulation of NVC in the spinal cord. This finding is consistent with the contribution of astrocytes to NVC reported for the cerebral cortex [2, 51]. This also suggests that although the mechanisms of blood flow regulation are different between the brain and the spinal cord, astrocytes seem to play a similar role in maintaining a tight relationship between changes in neuronal activity and blood flow.

### Effects of astrocyte inhibition on blood flow responses and neuronal activity

In the present study, noxious stimuli of relatively long duration (10 s) were used. The results indicate that astrocyte inhibition by fluorocitrate decreased SCBF responses to these stimuli. However, it is not clear if this applies to stimuli of shorter duration. Indeed, stimulus-evoked calcium changes in astrocytes may occur 3–4 s after stimulus onset in the visual cortex [35]. This suggests that astrocytes do not initiate the NVC and the stimulus-evoked blood flow responses [5, 44]. Accordingly, localized vascular responses in the barrel cortex occur without astrocyte activation during a 5-s whisker stimulation protocol [36]. Consistent with this idea, blood flow responses evoked by stimuli of relatively short duration may be less affected or not affected at all by astrocyte inhibition in the spinal cord so the present results may not be generalizable to noxious stimuli of shorter duration.

It should be noted that SCBF responses were not completely abolished by astrocyte inhibition, as expected. Indeed, although the astrocytes are a key component of the neurovascular unit, they are not the sole communication pathway between neurons and blood vessels [4]. Changes in extracellular K ^+^ generated by neuronal activity hyperpolarize capillary endothelial cells, which produces vasodilation of arterioles [20]. In the cortex, PGE2 released by pyramidal cells and NO released by NOS interneurons also contribute to the NVC [4]. It remains unclear whether this applies to the spinal cord, but it has been shown that NOS interneurons are present in high concentration in the dorsal horn of the spinal cord [46]. Thus, the direct communication between neurons and endothelial cells [25] and NOS interneurons may contribute to residual SCBF responses after astrocyte inhibition.

In addition to their vascular function, astrocytes provide neurons with nutriment such as lactate [37] and they contribute to the uptake and release of glutamate/glutamine [22]. Astrocyte inhibition by fluorocitrate also reduces these functions, which may in turn affect neuronal function. However, this is unlikely to affect the present results since LFP amplitude was stable over the 4 h of recording and was not significantly different between the fluorocitrate and saline groups. Moreover, previous studies indicate that fluorocitrate does not produce neuronal damage up to 24 h after its administration if it is not associated with neuronal stressors such as spreading depression [18]. In the present study, stimuli produced phasic neuronal activity and were applied with a relatively long inter-stimulus interval (55 s). This prevents tonic activity that may lead to neuronal stress as in spreading depression. In addition, histological examination of neurons after 4 h of fluorocitrate exposure has shown no damage, which became observable only after 8 h [16].

### Progressive astrocyte inhibition by fluorocitrate

Astrocytes uptake fluorocitrate selectively [6, 26, 49]. In the astrocyte, fluorocitrate blocks the Krebs cycle, which alters the production of ATP [31] and gradually decreases metabolic activity. This progressive inhibition of astrocytes by fluorocitrate was reported previously [9, 29]. In the present study, the inhibition of astrocytes was reflected in a progressive decrease in SCBF responses over time. In comparison to control SCBF responses, SCBF responses decreased by 23.8% one hour after fluorocitrate administration. The decrease in SCBF responses peaked after 3 h (40.2%) and was similar after 4 h (38.3%). This gradual effect is in agreement with the decrease of glutamine (only synthesized by astrocytes) 30 min after fluorocitrate treatment and the lowest level of glutamine at 3 h [16]. Similarly, fluorocitrate administration produces time-dependent changes of cAMP in astrocytes (peak of 94% at 2 h) [39] and in amino acid, with a maximal effect between 2 and 4 h [29].

### Limitation and further direction

In the present study, the conclusion on astrocyte inhibition by fluorocitrate relies on its effect on SCBF responses and the lack of effect on neuronal activity. Although the time course of the effect of fluorocitrate on SCBF is consistent with its expected effects on astrocytes [16, 29], with no effect on neuronal activity, future studies are needed to confirm astrocyte inhibition with a direct measure of astrocyte activity. Alternatively, astrocyte activity may be manipulated specifically with chemogenetic methods [10, 14].

Another limitation to consider is that pericytes contribute to the NVC in the brain [30] (review [40]). The present results may thus be explained by an indirect effect of fluorocitrate on pericytes through astrocyte inhibition since astrocytes partly regulate blood vessel diameter through communication with pericytes [40]. Nonetheless, it remains that the decreased SCBF responses are unlikely to be produced by a direct effect of fluorocitrate on pericytes.

## Conclusions

In conclusion, the present study indicates that astrocytes contribute to the regulation of NVC in the spinal cord. These results have implications for the use of spinal fMRI for clinical conditions in which astrocyte functions are altered, including amyotrophic lateral sclerosis [32].

## Data Availability

The datasets used and/or analyzed during the current study are available from the corresponding author on reasonable request.

## Abbreviations

LFP: Local field potentials
MAP: Mean arterial pressure
NVC: Neurovascular coupling
SCBF: Spinal cord blood flow
